# Enrichment of GABA_A_ Receptor α-Subunits on the Axonal Initial Segment Shows Regional Differences

**DOI:** 10.3389/fncel.2016.00039

**Published:** 2016-03-01

**Authors:** Yudong Gao, Scott A. Heldt

**Affiliations:** Department of Anatomy and Neurobiology, The University of Tennessee Health Science Center, MemphisTN, USA

**Keywords:** GABA_A_R, ankyrin G, amygdala, axonal initial segment, gephyrin

## Abstract

Although it is generally recognized that certain α-subunits of γ-aminobutyric acid type A receptors (GABA_A_Rs) form enriched clusters on the axonal initial segment (AIS), the degree to which these clusters vary in different brain areas is not well known. In the current study, we quantified the density, size, and enrichment ratio of fluorescently labeled α1-, α2-, or α3-subunits aggregates co-localized with the AIS-marker ankyrin G and compared them to aggregates in non-AIS locations among different brain areas including hippocampal subfields, basal lateral amygdala (BLA), prefrontal cortex (PFC), and sensory cortex (CTX). We found regional differences in the enrichment of GABA_A_R α-subunits on the AIS. Significant enrichment was identified in the CA3 of hippocampus for α1-subunits, in the CA1, CA3, and BLA for α2-subunits, and in the BLA for α3-subunits. Using α-subunit knock-out (KO) mice, we found that BLA enrichment of α2- and α3-subunits were physiologically independent of each other, as the enrichment of one subunit was unaffected by the genomic deletion of the other. To further investigate the unique pattern of α-subunit enrichment in the BLA, we examined the association of α2- and α3-subunits with the presynaptic vesicular GABA transporter (vGAT) and the anchoring protein gephyrin (Geph). As expected, both α2- and α3-subunits on the AIS within the BLA received prominent GABAergic innervation from vGAT-positive terminals. Further, we found that the association of α2- and α3-subunits with Geph was weaker in AIS versus non-AIS locations, suggesting that Geph might be playing a lesser role in the enrichment of α2- and α3-subunits on the AIS. Overall, these observations suggest that GABA_A_Rs on the AIS differ in subunit composition across brain regions. As with somatodendritic GABA_A_Rs, the distinctive expression pattern of AIS-located GABA_A_R α-subunits in the BLA, and other brain areas, likely contribute to unique forms of GABAergic inhibitory transmission and pharmacological profiles seen in different brain areas.

## Introduction

Gamma-amino butyric acid (GABA) is the main inhibitory neurotransmitter in the central nervous system and acts at synapses through binding to ligand-gated the GABA type A receptors (GABA_A_Rs). In principal neurons, postsynaptic GABA_A_Rs localized on synaptic and extrasynaptic membranes mediate phasic and tonic inhibition, respectively (Farrant and Nusser, 2005). Many of these postsynaptic receptors form symmetric synapses on the shaft of dendrites or around the somata of a neurons. In addition to these somatodendritic locations, GABA_A_Rs are also located on the axon initial segment (AIS) of principal neurons where they are clustered in rows of synapses and targeted almost exclusively by inputs from chandelier interneurons (Soriano et al., 1990; Freund and Buzsaki, 1996; Woodruff et al., 2009; Inan and Anderson, 2014; Wefelmeyer et al., 2015). The AIS is a crucial subcellular domain on neurons where action potentials are initiated, and plays a pivotal role in neuronal excitability (Stuart et al., 1997; Kole et al., 2008). Besides GABA_A_Rs, ion channels, such as voltage-gated sodium channels (NaV channels) and certain types of potassium channels (KCNQ channels), are known to form clusters on the AIS via a protein-protein interaction mechanism with ankyrin G (AnkG), an anchoring protein that links them with spectrin-actin cytoskeletal scaffolds around the AIS (Pan et al., 2006). It has been postulated that the AIS-located GABA_A_Rs may provide chandelier cartridges with a powerful inhibitory action on the output of principal neurons (Glickfeld et al., 2009). However, a predominantly depolarizing effect upon activation of AIS GABA_A_Rs has been shown in various brain regions, including pyramidal neurons in the neocortex (Szabadics et al., 2006), amygdala (Woodruff et al., 2006), and hippocampus (Khirug et al., 2008).

Previous studies have shown that clusters of GABA_A_Rs containing the α2-subunits are preferentially enriched at AIS synaptic sites of hippocampus and cortex compared to those containing the α1-subunits (Nusser et al., 1996; Fritschy et al., 1998a; Loup et al., 1998; Nyíri et al., 2001; Brünig et al., 2002; Tretter et al., 2008; Panzanelli et al., 2011; Muir and Kittler, 2014). Immunohistochemical and immunocytochemical studies have also identified AIS-located GABA_A_Rs containing the α3-subunit in the cortex and cultured hippocampal neurons (Fritschy et al., 1998a; Loup et al., 1998; Brünig et al., 2002), however, the extent to which they are enriched relative to α1- and α2-subunits is unknown. Because GABA_A_R subtypes differing in α-subunit composition have distinct physiological and pharmacological properties, knowing the differential distribution of AIS-located α-subunits will likely help explain the unique forms of GABAergic inhibitory transmission and pharmacological profiles seen in different brain areas. GABA_A_Rs containing the α1–α3 subunits also differ in their dependence on signaling molecules involved in synapse-specific anchoring and stabilizing of GABA_A_Rs at postsynaptic sites, including gephyrin (Geph), an anchoring protein (Jacob et al., 2008; Tretter et al., 2008, 2012; Mukherjee et al., 2011). Thus, knowing the relationship between AIS-located GABA_A_R clusters and Geph may help the understanding of the cellular mechanisms that control subcellular targeting of postsynaptic GABA_A_Rs.

In this study, we investigated the differential expression pattern of α1-, α2-, and α3-subunits on the AIS across six different brain areas, namely the CA1, CA3 and dentate gyrus (DG) of the hippocampus, the basal lateral amygdala (BLA), the prefrontal cortex (PFC), and the cortex (CTX). We also investigated to what degree do the enriched GABA_A_R α-subunits on the AIS associate with Geph. We found the expression pattern of α1-, α2-, and α3-subunits in the AIS differed across different brain areas, with enrichment on the AIS identified in the CA3 of hippocampus for α1-subunits, in the CA1, CA3 and BLA for the α2-subunits, and in the BLA for the α3-subunits. We also found that the AIS enrichment of α2- and α3-subunits in the BLA is physiologically independent of each other, as the enrichment of one subunit is unaffected by the genomic knock-out (KO) of the other.

Further, we investigated the cellular properties of the AIS-located GABA_A_R subunits. We found that the α2- and α3-subunits on the AIS receive heavy innervation from and are closely coupled with the vesicular GABA transporter (vGAT)-positive terminals. To our surprise, the association of those AIS-located α2- and α3-subunits with Geph was found to be much weaker than in non-AIS locations, suggesting that Geph might be playing a lesser role in the clustering and enrichment of α2- and α3-subunits on the AIS.

## Materials and Methods

### Subjects

Male C57BL/6J mice were purchased from Jackson laboratory and group housed in microisolation cages at the University of Tennessee Health Science Center animal facility, with *ad libitum* access to food (Teklad rodent diet, Harlan Laboratories, Indianapolis, IN, USA) and water. The facility also provided a 12 h light:dark cycle and controlled temperature/humidity. All protocols were approved by the Animal Care and Use Committee of the University of Tennessee Health Science Center in accord with principles and standards of animal care outlined by the National Institute of Health Institutional Animal Care and Use Committee. Fresh frozen brain specimens of mice lacking the GABA_A_R α2-subunit (α2-KO) or GABA_A_R α3-subunit (α3-KO) were kindly provided by Dr. Uwe Rudolph, from McLean Hospital. These strains were originally described in previous publications (Yee et al., 2005; Witschi et al., 2011).

### Immunohistochemistry

In line with a previous study (Tian et al., 2014), pilot experiments revealed some AIS-located proteins were sensitive to fixation conditions. Tissue fixation carried out by traditional perfusion and cryoprotection in 4% paraformaldehyde yielded weak AIS immunelabeling of both AnkG and GABA_A_R α-subunits (data not shown). On the other hand, brief fixation of fresh frozen sections in ice cold 4% paraformaldehyde solution yielded both reliable fixation strength, and consistent AIS immunolabeling signals. Thus, the latter tissue processing method was used in this study. Brains were rapidly isolated and immediately frozen over crushed dry ice. The fresh frozen brains were imbedded in Tissue-Tek O.C.T. compound (Sakura Finetek, Torrance, CA, USA), coronally sectioned at a thickness of 7–10 μm on a Leica 3050S cryostat, (Leica Biosystems Inc., Buffalo Grove, IL, USA) and mounted on Superfrost Plus slides (Fisher Scientific, Pittsburgh, PA, USA). Sections were briefly fixed with ice cold 4% paraformaldehyde in phosphate buffered saline solution (PBS) for 15 min. After three washes in 0.1 M phosphate buffer (PB), slides were incubated with a blocking buffer containing 0.2% saponin, 0.1% tween 20, and 2% normal goat serum in PB for 45 min under room temperature. Combinations of primary antibodies (listed in **Table 1**) were diluted in blocking buffer and incubated with the specimen overnight at 4°C. After three washes in blocking buffer, combinations of Alexa-conjugated secondary antibodies corresponding to the primary antibody species (listed in **Table 1**) were applied and incubated for 1 h under room temperature. After three washes in blocking buffer, slides were rinsed with tap water, air dried and coverslipped with VECTASHIELD HardSet DAPI mounting medium (Vector Laboratories, Burlingame, CA, USA) for further microscopic examination. For three dimensional (3D) reconstruction experiment, 30 μm fresh frozen sections were collected on Superfrost Plus slides and the same immunohistochemistry procedures described above were carried out.

**Table 1:** List of primary and secondary antibodies used in this study.

Primary antibodies	Manufacturer	Cat #	Dilution
Rabbit anti-α1	Alomone Labs	AGA-001	1:500
Rabbit anti-α2	Synaptic Systems	224 103	1:500
Rabbit anti-α3	Alomone Labs	AGA-003	1:500
Mouse anti-AnkG	Invitrogen	33-8800	1:500
Mouse anti-Geph	Synaptic Systems	147 011	1:300
Guinea pig anti-vGAT	Synaptic Systems	131 004	1:500

**Secondary antibodies**	**Manufacturer**	**Cat #**	**Dilution**

Alexa488 goat anti-rabbit	Invitrogen	A-11008	1:1000
Alexa568 goat anti-mouse	Invitrogen	A-11004	1:1000
Alexa488 goat anti-guinea pig	Invitrogen	A-11073	1:1000
Alexa647 donkey anti-mouse	Invitrogen	A-31571	1:1000
Alexa568 goat anti-rabbit	Invitrogen	A-11011	1:1000

### Image Acquisition

**FIGURE 1:**
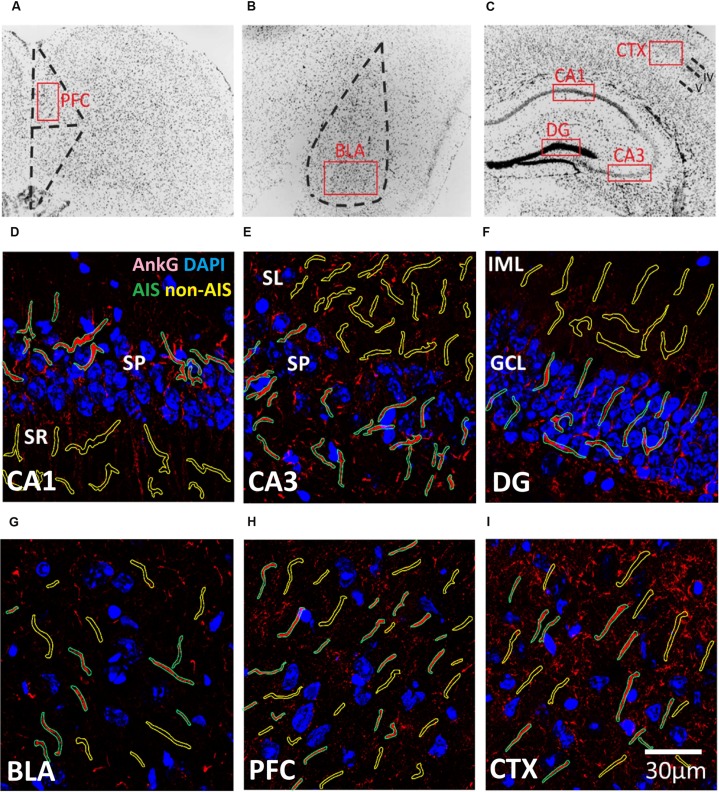
**(A–C)** Representative illustration of the brain areas where immunofluorescent images were acquired from the **(A)** prefrontal cortex (PFC), **(B)** basal lateral amygdala (BLA), and **(C)** CXT/hippocampal regions (CA1, CA3, and DG). Areas were first located under 10× objective lens based on the contours of DAPI staining, and high magnification images were subsequently acquired using a 63× objective lens within the boundaries of each brain area (red boxes). **(D–I)** Examples of AIS (green) and Non-AIS (yellow) ROI contours delineated based on the AnkG (red) and DAPI (blue) confocal images of the **(D)** CA1, **(E)** CA3, **(F)** DG, **(G)** BLA, **(H)** PFC, and **(I)** CTX. Abbreviations: DG, dentate gyrus; BLA, basal lateral amygdala; PFC, prefrontal cortex; CXT, somatosensory barrel cortex; SP, stratum pyramidale; SR, stratum radiatum; SL, stratum lucidum; IML, inner molecular layer; GCL, granule cell layer.

Confocal imaging was performed using Zeiss LSM 710 confocal microscopy system (Carl Zeiss Microscopy GmbH, Jena, Germany). Brain area were identified from coronal sections at the following approximate center locations in reference to Bregma according to the following stereotaxic coordinates (Paxinos and Franklin, 2001): CA1: AP -1.6 mm, ML ± 1.0 mm, DV -1.5 mm; CA3: AP -1.6 mm, ML ± 1.5 mm, DV -2.2 mm; DG: AP -1.6 mm, ML ± 0.9 mm, DV -2.0 mm; BLA: AP -1.6 mm, ML ± 3.1 mm DV -4.8 mm, PFC: AP: 2.5 mm, ML ± 0.2 mm, DV -1.5 mm. CTX: AP -1.6 mm, ML ± 2.2 mm, DV -1.1 mm (**Figure 1**). Images of the CTX were approximately centered layers 4 and 5 of the barrel cortex. Images of the PFC were centered in the pre-limbic area, approximately layer 3. Immunofluorescence images of the immunoreactivity were acquired from selected brain regions with a Plan-Apochromat 63x/1.40 Oil DIC objective lens with the pinhole set at 1 airy unit (1 AU). Laser power and gain were manually adjusted for each exposure to minimize saturation while maintaining satisfactory signal intensities and dynamic ranges. This contributes to the semi-quantitative nature of the analysis and the result should not be interpreted as the absolute values of subunit expression. Each image (2000 pixels × 2000 pixels) represented a single optical section and each pixel covers ∼64 nm of the specimen. For 3D reconstruction experiments, the pinhole was set at 0.5 AU and z-stack image series (600 pixels × 600 pixels) were collected for subsequent processing on Imaris workstation (Bitplane AG, Zurich, Switzerland).

### Image Processing

Results were obtained from a pairs of images acquired from the same optical plane within the amygdalar, cortical, or hippocampal brain regions collected from three mice. Image processing for each statistical analysis was the same for all brain regions and slides. All images were background subtracted using a rolling ball algorithm (radius 50 pixels). Inspection of immunoreactivity signal histograms of GABA_A_R α-subunits and Geph images revealed maximum peak gray levels below 50. Thus, to identify the outline of α-subunit and Geph aggregates, images were binarized using the triangle threshold algorithm, which is an accepted method for images whose histogram has a maximum near one of the extremes (e.g., Zack et al., 1977). The density, surface area size, and percent area of α-subunit and Geph aggregates (puncta-like clusters) were automatically identified and quantified in defined regions of interest (ROIs, described below) using the Analyze Particles algorithm included in the open source image processing program Fiji (Schindelin et al., 2012; Schneider et al., 2012). The percent area denoted the percentage of the ROI area that contained identified clusters. The Analyze Particles algorithm was configured to identify areas of positive aggregates between 0.05 and 10.00 μm^2^ with circularity of 0.10 to 1.00. If no puncta were detected within a ROI, the cluster density was set to 0 and the cluster size was set to missing value.

To analyze the expression of α-subunits on the AIS of C57BL/6J mice, AIS regions were delineated manually as ROIs following the contour of positive AnkG staining, a selective marker of the AIS (Rasband, 2010). Non-AIS regions, with contours identical to AIS regions, were also delineated as ROIs. These latter regions lacked both identifiable AnkG staining and cytoplasmic structures. Non-AIS regions were located in the inner molecular layer of DG, stratum radiatum of the CA1, stratum lucidum of the CA3, or layers 4 and 5 of the cortex. A total of 43–69 AIS and non-AIS ROIs were sampled from 2 to 3 images taken from brain areas of three mice. To analyze the expression of α-subunits on the AIS of α2-KO and α3-KO mice, similar procedures were carried out. A total of 20–32 AIS and non-AIS ROIs were sampled from 2 to 3 images taken from each brain area of a α2-KO or α3-KO mouse.

To investigate the association of Geph with selected α-subunits, we quantified the correlation between Geph and α-subunit immunoreactivity intensities using Pearson’s coefficient (Costes et al., 2004) and the overlap of aggregates (co-localization) using Mander’s coefficients (Manders et al., 1993). For correlations, non-binarized images were subjected to auto-thresholding, followed by co-localization quantification (Costes et al., 2004; Zinchuk and Grossenbacher-Zinchuk, 2009). Co-localization measurements were derived from binarized images and restricted to AIS and non-AIS ROIs. AIS ROIs were delineated manually by following the contour of GABA_A_R aggregates arranged in a “beads along a string” pattern, a characteristic of AIS α-subunit clusters. Applying this method to single-channel images of α1–α3 GABA_A_R subunits co-labeled with AnkG revealed that the proportion of delineated α2- and α3-subunits overlapping with AnkG expression was reliably high in the BLA (α2: 18/20 = 90%; α3: 19/20 = 95%). This method also dependably identified overlapping α2-subunits with AnkG expression in the CA3 (α2: 22/24 = 92%). The probability of identifying overlapped signals was notably lower in other comparisons, so we limited our analysis to these AIS clusters. Non-AIS regions, with contours identical to AIS regions were also defined as ROIs. Both Pearson’s and Mander’s coefficients were computed using the Coloc 2 plugin in Fiji to quantify co-localization (Dunn et al., 2011; Schindelin et al., 2012; Adler and Parmryd, 2013; Pompey et al., 2013). A total of 37–44 AIS and non-AIS ROIs were sampled from 2 to 3 images taken in each brain area of three mice.

### Statistical Analyses

Data were expressed as means ± standard errors of the mean (SEM). Statistical procedures were carried out using JMP Pro version 10 (SAS Institute, Inc., Cary, NC, USA) and SPSS version 21 (IBM, Armonk, NY, USA). Two-way ANOVAs were used to assess differences in the mean density and mean size of clusters using location (AIS, non-AIS) and brain area (CA1, CA3, DG, BLA, PFC, CTX) as factors. These means were calculated by averaging ROI measurements acquired from all images. Significant main effects and interactions were evaluated with one-way ANOVAs and/or Bonferroni planned multiple comparison method. To identify and compare differences in the AIS aggregation of clusters among brain areas, we computed “enrichment ratios,” which took into account differences in the density and size of clusters between areas. These ratios were calculated by dividing the mean percent area of AIS clusters by the mean percent area of non-AIS clusters. As mentioned above, non-AIS ROIs lacked identifiable somatic structures, thus these ratios estimate the enrichment of AIS clusters relative to areas containing dendritic segments and distal axons of principle cells and inhibitory interneurons. One-way ANOVAs were used to assess differences in the enrichment ratios between brain areas. Significant main effects were further evaluated by *post hoc* pair-wise multiple comparisons using Tukey-Kramer method. To investigate the association of Geph with selected α-subunits, Pearson’s and Mander’s coefficients, namely the *R*-value and the *M*1/*M*2-values, were compared between the AIS and non-AIS locations by Bonferroni corrected multiple *t*-tests comparisons.

## Results

### Co-labeling of AnkG and GABA_A_R α-Subunits in C57BL/6J Mice

Previous studies have shown that clusters of GABA_A_Rs containing the α2-subunits are preferentially targeted to the AIS compared to those containing the α1-subunits (Nusser et al., 1996). To describe the expression patterns of AIS α3-subunits in comparison to α1- and α2-subunits, we quantified and compared the density, size, and enrichment ratio of subunit aggregates in the AIS and non-AIS ROIs both within and across brain regions as described below.

#### Co-labeling of AnkG and α1-Subunits

**FIGURE 2:**
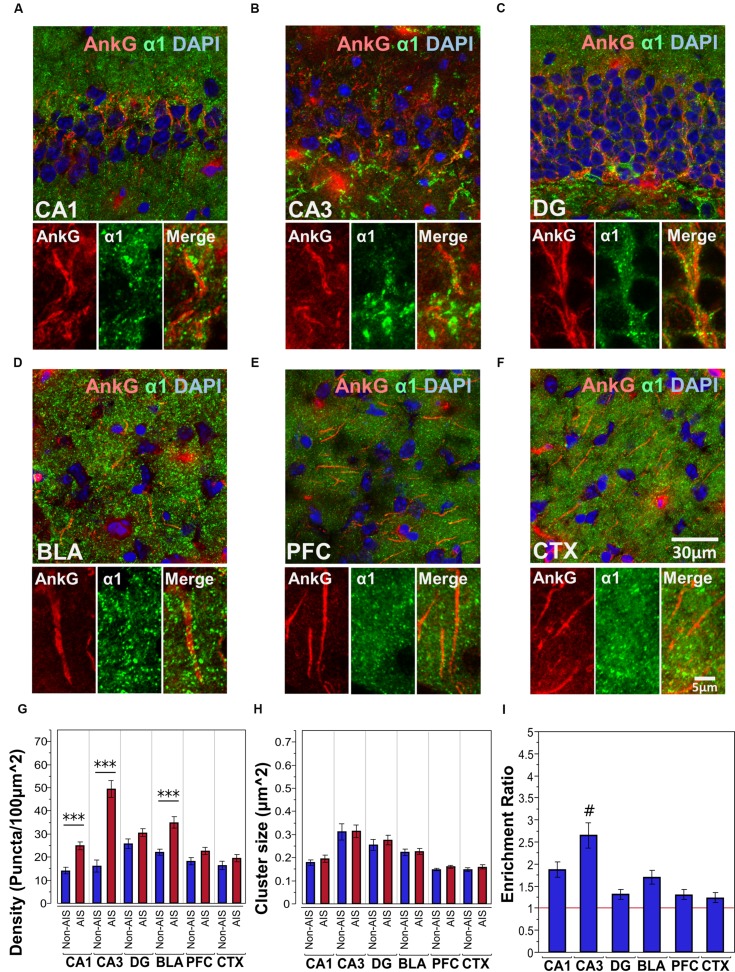
**(A–F)** Immunofluorescent double-labeled montage images of AnkG (red) and α1-subunits (green) taken from single optical sections of the hippocampal sub-regions (CA1, CA3, DG), BLA, PFC, and CTX in C57BL/6J mice. **(G,H)** Average cluster density and size of α1-subunits on the AIS and non-AIS locations were reported as means ± SEM. Significant differences of cluster density were identified in the CA1, CA3, and the BLA, while no statistical significant difference was observed for the cluster size. Asterisks represent significant difference from the AIS and the non-AIS locations using the Bonferroni method for planned multiple comparison, ^∗∗∗^*p* < 0.001. **(I)** Enrichment ratios of α1-subunits across different brain areas were reported. Tukey-Kramer *post hoc* pair-wise multiple comparison revealed the enrichment ratio of α1-subunits in the CA3 was significantly higher than the other brain areas, #*p* < 0.05.

**Figures 2A–F** shows immunofluorescent double-labeled montage images of AnkG and α1-subunits taken from single optical sections of the hippocampal sub-regions (CA1, CA3, DG), BLA, PFC, and CTX. The mean densities, sizes, and enrichment ratios of α1-subunit clusters are presented in **Figures 2G–I**. Analysis of α1-subunit cluster densities revealed significant main effects of location [*F*_(1,628)_ = 100.25, *p* < 0.001] and brain area [*F*_(5,628)_ = 17.84, *p* < 0.001], and a significant Location × Brain Area interaction [*F*_(5,628)_ = 14.88, *p* < 0.001]. Direct comparisons between locations in each brain area showed AIS-located densities were significantly higher than non-AIS densities in the CA1, CA3, and BLA (*p*s < 0.001, after Bonferroni correction). The analysis of α1-subunit cluster sizes revealed a significant main effect of brain area [*F*_(5,602)_ = 27.91, *p* < 0.001], but no significant effect of location or Location × Brain Area interaction (*p*s > 0.05). Direct comparisons between locations in each brain area also showed no differences regarding to cluster size. Assessment of enrichment ratios indicated significant differences in the AIS aggregation of α1-subunit clusters among brain areas [*F*_(5,315)_ = 10.07, *p* < 0.001]. *Post hoc* comparisons using Tukey-Kramer HSD method showed that the enrichment of α1-subunit clusters in the CA3 was significantly greater than in the CA1, DG, BLA, CTX, and PFC (*p*s < 0.05).

#### Co-labeling of AnkG and α2-Subunits

**FIGURE 3:**
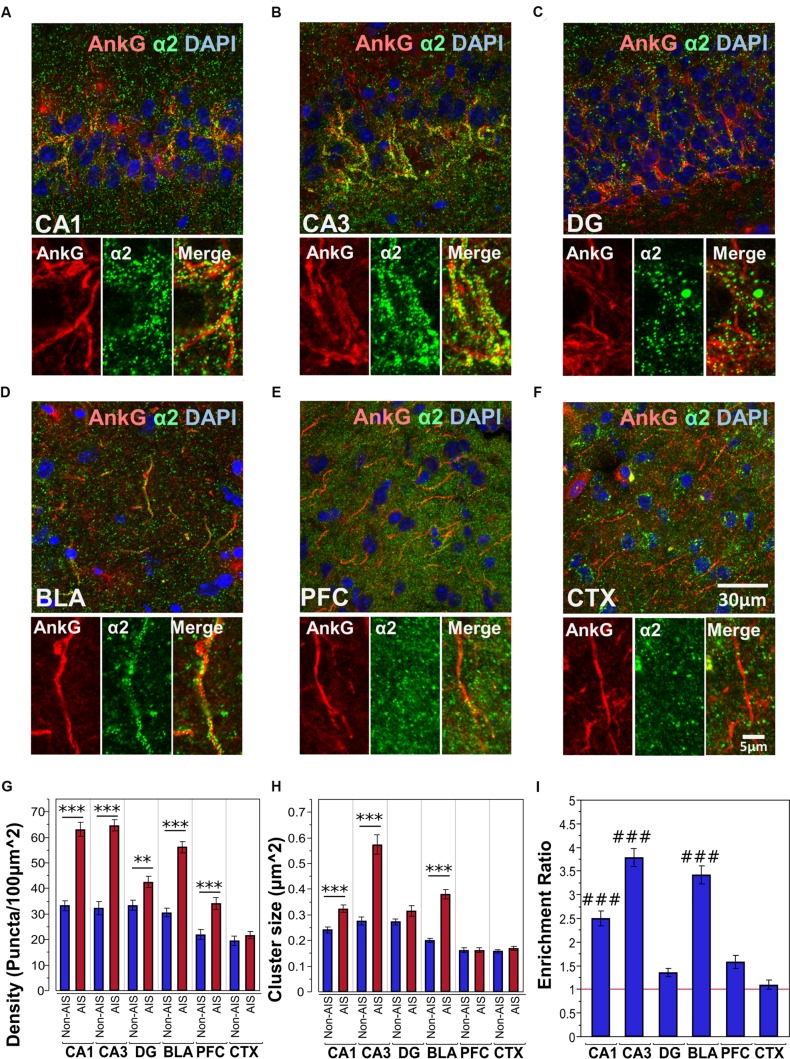
**(A–F)** Immunofluorescent double-labeled montage images of AnkG (red) and α2-subunits (green) taken from single optical sections of the hippocampal sub-regions (CA1, CA3, DG), BLA, PFC, and CTX in C57BL/6J mice. **(G,H)** Average cluster density and size of α2-subunits on the AIS and non-AIS locations were reported as means ± SEM. Significant differences of cluster density were identified in the CA1, CA3, DG, BLA, and PFC. Significant differences of cluster size were identified in the CA1, CA3, and BLA. Asterisks represent significant difference from the AIS and the non-AIS locations using the Bonferroni method for planned multiple comparison, ^∗∗^*p* < 0.01, ^∗∗∗^*p* < 0.001. **(I)** Enrichment ratios of α2-subunits across different brain areas were reported. Tukey-Kramer *post hoc* pair-wise multiple comparison revealed the enrichment ratios of α2-subunits in the CA1, CA3, and BLA were significantly higher than the other brain areas, ###*p* < 0.001.

**Figures 3A–F** shows immunofluorescent double-labeled montage images of AnkG and α2-subunits taken from single optical sections of the hippocampal sub-regions (CA1, CA3, DG), BLA, PFC and CTX. The mean densities, sizes, and enrichment ratios of α2-subunit clusters are presented in **Figures 3G–I**. Analysis of α2-subunit cluster densities revealed significant main effects of location [*F*_(1,718)_ = 224.13, *p* < 0.001] and brain area [*F*_(5,718)_ = 56.01, *p* < 0.001], as well as a significant Location × Brain Area interaction [*F*_(5,718)_ = 16.80, *p* < 0.001]. Direct comparisons between locations in each brain area showed AIS-located densities were significantly higher than non-AIS densities in the CA1, CA3, DG, BLA and PFC (*p*s < 0.01, after Bonferroni correction for planned multiple comparisons). The analysis of α2-subunit cluster sizes revealed significant main effects of location [*F*_(1,706)_ = 115.05, *p* < 0.001] and brain area [*F*_(5,706)_ = 69.07, *p* < 0.001], as well as a significant Location × Brain Area interaction [*F*_(5,706)_ = 24.06, *p* < 0.001]. Direct comparisons between locations in each brain area showed AIS-located α2-subunit cluster sizes were significantly larger than non-AIS clusters in the CA1, CA3, and BLA (*p*s < 0.001, after Bonferroni correction). Assessment of enrichment ratios indicated significant differences in the AIS aggregation of α2-subunit clusters among brain areas [*F*_(5,359)_ = 58.13, *p* < 0.001]. *Post hoc* pair-wise comparisons showed that the enrichment of α2-subunit clusters in the CA1, CA3, BLA was significantly greater than in the DG, CTX, and PFC (*p*s < 0.01).

#### Co-labeling of AnkG and α3-Subunits

**FIGURE 4:**
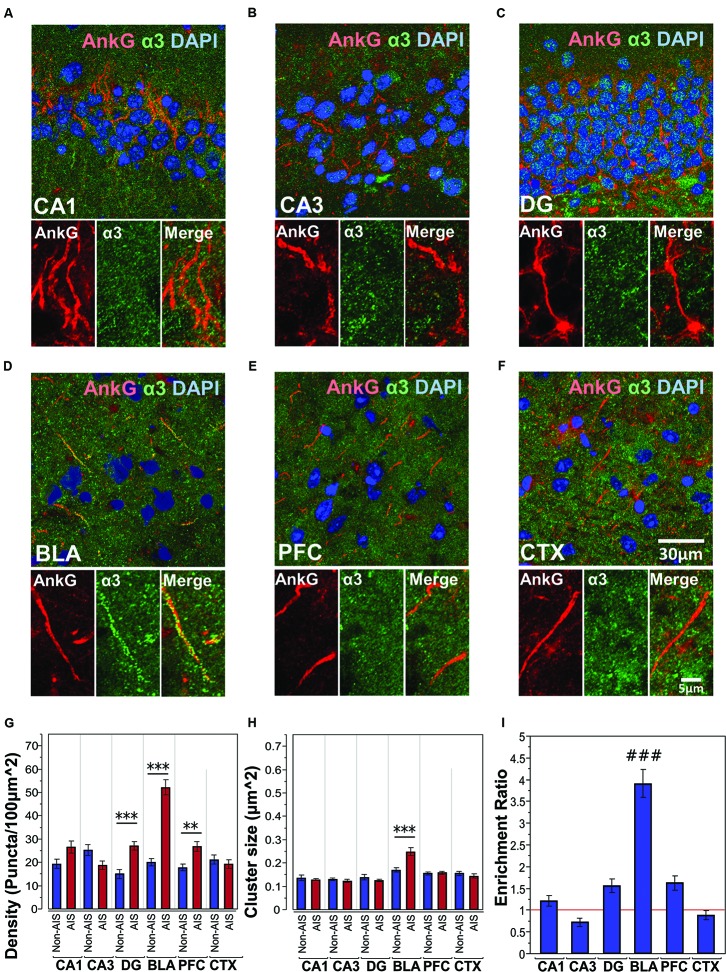
**(A–F)** Immunofluorescent double-labeled montage images of AnkG (red) and α3-subunits (green) taken from single optical sections of the hippocampal sub-regions (CA1, CA3, DG), BLA, PFC, and CTX in C57BL/6J mice. **(G,H)** Average cluster density and size of α3-subunits on the AIS and non-AIS locations were reported as means ± SEM. Significant differences of cluster density were identified in the DG, BLA, and PFC. Significant difference of cluster size was identified in the BLA. Asterisks represent significant difference from the AIS and the non-AIS locations using the Bonferroni method for planned multiple comparison, ^∗∗^*p* < 0.01, ^∗∗∗^*p* < 0.001. **(I)** Enrichment ratios of α3-subunits across different brain areas were reported. Tukey-Kramer *post hoc* pair-wise multiple comparison revealed the enrichment ratio of α3-subunits in the BLA was significantly higher than the other brain areas, ###*p* < 0.001.

**Figures 4A–F** shows immunofluorescent double-labeled montage images of AnkG and α3-subunits taken from single optical sections of the hippocampal sub-regions (CA1, CA3, DG), BLA, PFC, and CTX. The mean densities, sizes, and enrichment ratios of α3-subunit clusters are presented in **Figures 4G–I**. Analysis of α3-subunit cluster densities revealed significant main effects of location [*F*_(1,637)_ = 51.33, *p* < 0.001] and brain area [*F*_(5,637)_ = 16.13, *p* < 0.001], as well as a significant Location × Brain Area interaction [*F*_(5,637)_ = 20.70, *p* < 0.001]. Direct comparisons between locations in each brain area showed AIS-located densities were significantly higher than non-AIS densities in the DG, BLA, and PFC (*p*s < 0.01, after Bonferroni). The analysis of α3-subunit cluster sizes revealed significant main effect of brain area [*F*_(5,610)_ = 23.66, *p* < 0.001] and Location × Brain Area interaction [*F*_(5,610)_ = 7.79, *p* < 0.001]. No significant effect of location was detected [*F*_(1,610)_ = 1.77, *p* = 0.184]. Direct comparisons of locations in each brain area showed that the size α3-subunit AIS cluster were significantly larger than non-AIS clusters in the BLA (*p* < 0.001, Bonferroni correction). Assessment of enrichment ratios indicated significant differences in the AIS aggregation of α3-subunit clusters among brain areas [*F*_(5,318)_ = 43.61, *p* < 0.001]. *Post hoc* pair-wise comparisons showed the enrichment of α3-subunit AIS clusters in the BLA was significantly greater than in all other brain areas (*p*s < 0.001).

### Co-labeling of AnkG and GABA_A_R α-Subunits in the BLA of α2- and α3-KO Mice

**FIGURE 5:**
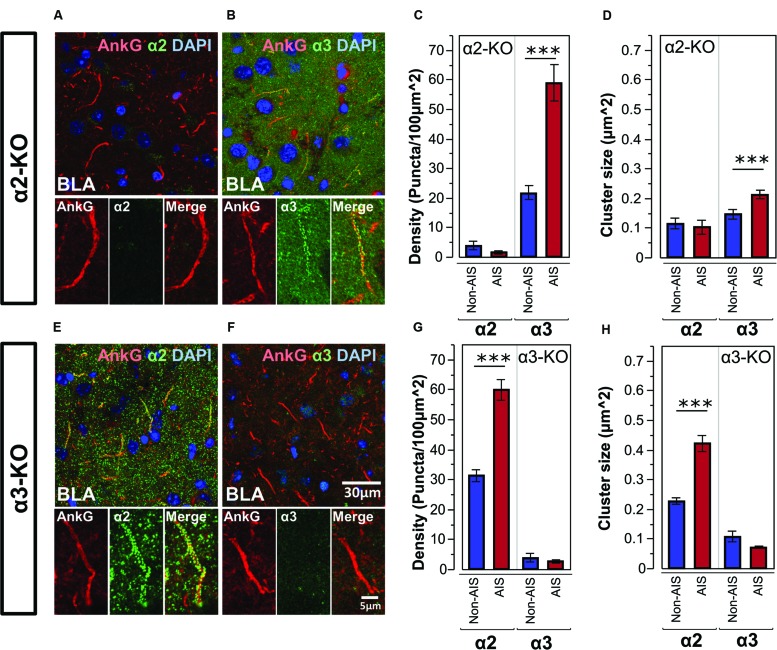
**(A,B)** Immunofluorescent double-labeled montage images of AnkG (red) and α2- and α3-subunits (green) taken from single optical sections of the BLA in α2-KO mouse. **(C,D)** Average cluster density and size of the α2- and α3-subunits on the AIS and non-AIS locations in α2-KO were reported as means ± SEM. Asterisks represent significant difference from the AIS and the non-AIS locations using the Bonferroni method for planned multiple comparison, ^∗∗∗^*p* < 0.001. **(E,F)** Immunofluorescent double-labeled montage images of AnkG (red) and α2- and α3-subunits (green) taken from single optical sections of the BLA in α3-KO mouse. **(G,H)** Average cluster density and size of the α2- and α3-subunits on the AIS and non-AIS locations in α3-KO were reported as means ± SEM. Asterisks represent significant difference from the AIS and the non-AIS locations using the Bonferroni method for planned multiple comparison, ^∗∗∗^*p* < 0.001.

**Figures 5A,B**, shows double-labeled montage images of AnkG and α2- or α3-subunits in the BLA taken from brain sections of α2-KO. In α2-KO, α2-subunit immunoreactivity was immensely reduced on AIS and non-AIS locations, corresponded to a loss of densities of roughly 97 and 87% in comparison to wild type (WT) C57BL/6J mice. In contrast, the density and size of α3-subunit AIS and non-AIS clusters in α2-KO were comparable to WT mice. Quantitative results of the cluster density and size of α3-subunits showed the loss of α2-subunits did not disrupt the enrichment of α3-subunits in the AIS, as shown in **Figures 5C,D**.

**Figures 5E,F**, shows double-labeled montage images of AnkG and α2- or α3-subunits in the BLA taken from brain sections of α3-KO. In α3-KO, α3-subunit immunoreactivity was immensely reduced on AIS and non-AIS locations, corresponded to a loss of densities of roughly 95% in the AIS and 80% in the non-AIS when compared to WT mice. In contrast, the density and size of α2-subunits clusters on AIS and non-AIS locations were comparable to WT mice. Quantitative results of the cluster density and size of α2-subunits showed the loss of α3-subunits did not disrupt the enrichment of α2-subunits in the AIS, as shown in **Figures 5G,H**.

### Tri-labeling of AnkG, vGAT, and α2- or α3-Subunits in the BLA and CA3 of C57BL/6J Mice

**FIGURE 6:**
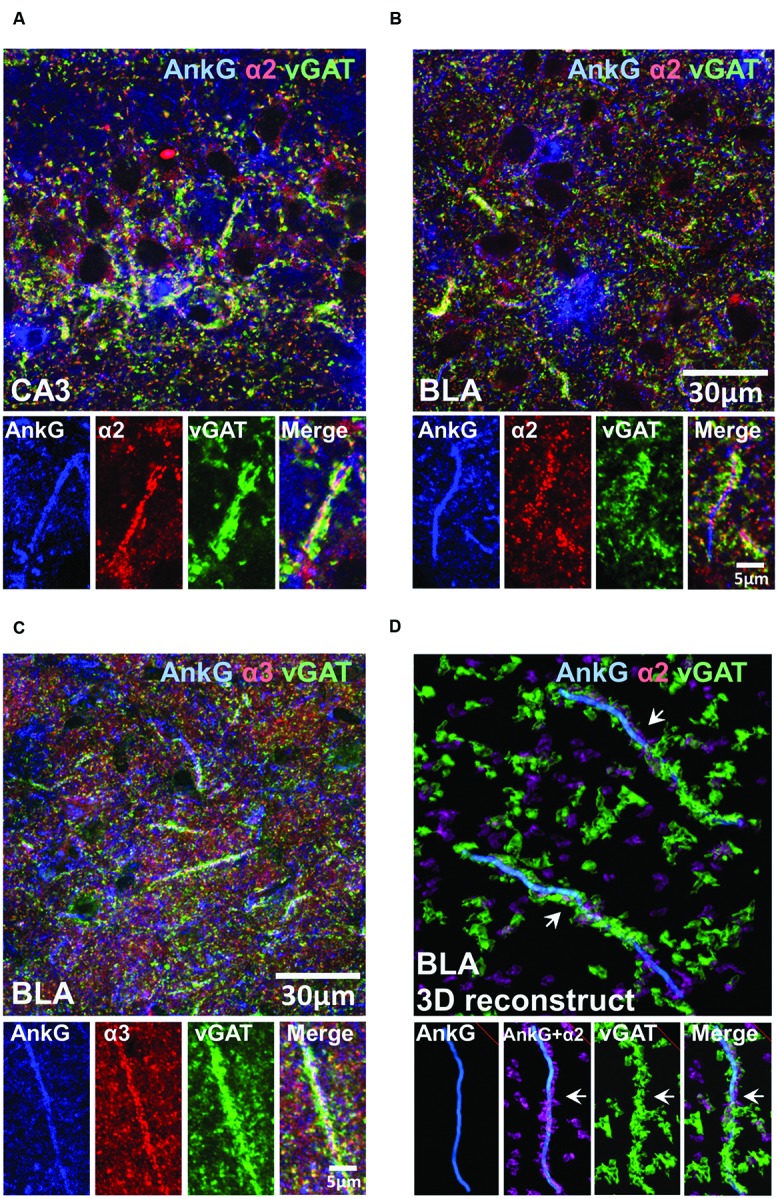
**(A–C)** Immunofluorescent triple-labeled montage images of AnkG (blue), α2/3-subunits (red), and vGAT (green) taken from single optical sections of the CA3 and BLA in C57BL/6J mice. Prominent triple-colocalizations in all images were evident. **(D)** 3D-reconstructed contours of AnkG, α2-subunit, and vGAT from z-stack image series taken in the BLA. The arrows indicated the turning points where the vGAT-positive terminals wrap around the AnkG-positive AIS.

To investigate whether the presence of enriched α2- or α3-subunits on the AIS was influenced by presynaptic GABAergic innervation, we performed tri-labeling of AnkG, vGAT, and α2- or α3-subunits. **Figures 6A–C** shows tri-labeled montage images of AnkG (blue), α-subunits (red), and vGAT (green) taken from sections of the CA3 and BLA. **Figure 6D** shows 3D-reconstructed image of AnkG, α2-subunit, and vGAT in the BLA. It was evident that vGAT-positive terminals opposed α2-subunit clusters in a spiraling path around the AIS. The arrows indicated the turning points where the vGAT terminals wrap around the AIS. Qualitative observations reveal evidence of strong triple co-localization, suggesting that AIS α2- and α3-subunits receives GABAergic innervation from vGAT-positive synaptic terminals.

### Association Geph and α2- or α3-Subunits on the AIS

**FIGURE 7:**
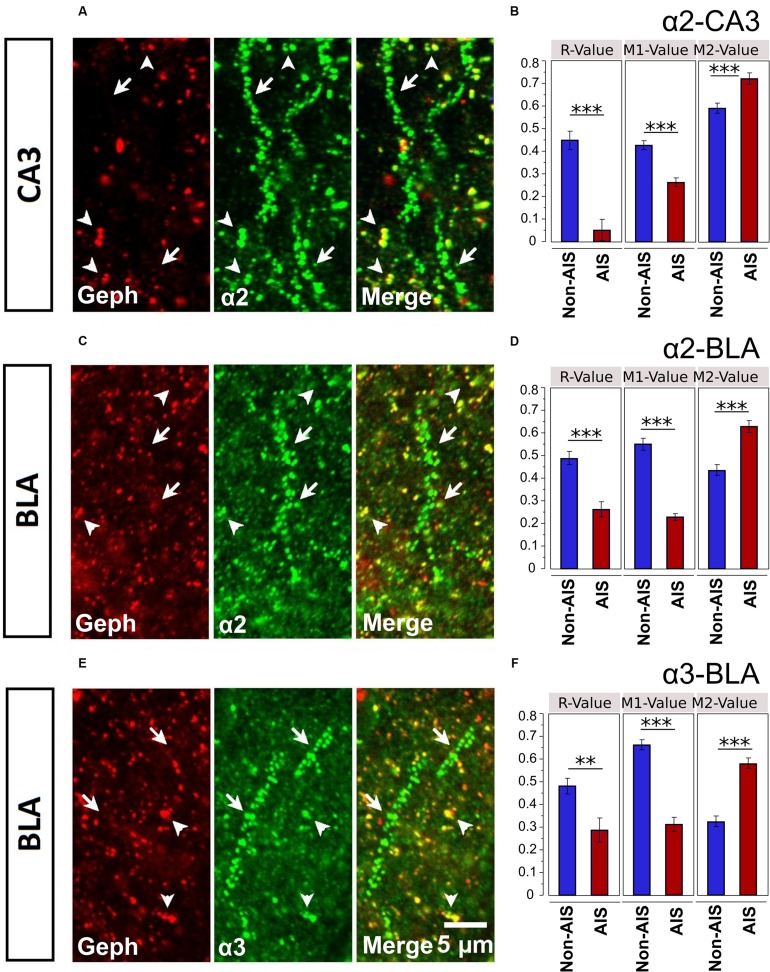
**(A,C,E)** Immunofluorescent double-labeled montage images of Geph (red), and α2/3-subunits (green) taken from single optical sections of the CA3 and BLA in C57BL/6J mice. Arrows with tails were pointed to the AIS regions, whereas the arrows without tails were pointed to the non-AIS regions. **(B,D,F)** Quantification results (Pearson’s *R*-value, and Manders’ *M*1 and *M*2 values) of the colocalizations of the α2- and α3-subunits with Geph on the AIS-like and non-AIS-like locations were reported as means ± SEM. Bonferroni-corrected planned multiple *t*-test comparisons revealed both the *R*-values and the *M*1-values (denoting the degree of α-subunit immunoreactivity that is overlapped with Geph immunoreactivity) were significantly lower in AIS-like locations when compared to non-AIS-like locations, whereas the M2-values (denoting the degree of Geph immunoreactivity that is overlapped with α-subunit immunoreactivity) were significantly higher in AIS-like locations when compared to non-AIS-like locations, ^∗∗^*p* < 0.01, ^∗∗∗^*p* < 0.001.

Geph is one of key players in the synaptic clustering of GABA_A_Rs; thus, to explore the possible role Geph plays in regulating the clustering of AIS α-subunits, we examined the relationship between Geph and α2- and α3-subunits clusters in AIS and non-AIS ROIs. In both the AIS and non-AIS regions of the CA3 and BLA, Geph formed discrete clusters, **Figures 7A,C,E**. Discrete aggregates were also located on the perisomatic membranes, however, in the absence of a fluorescent membrane dye, these clusters were difficult to clearly ascertain or quantify. As noted by others, we observed the accumulation of both large and small puncta within cytoplasmic areas of some neurons, as indicated by DAPI-staining (Sun et al., 2004; Poulopoulos et al., 2009; Muir and Kittler, 2014). We could also visualize Geph clusters that were not co-localized α2- or α3-subunits, presumably associated with other α-subunits or glycine receptors.

Double-immunofluorescence staining in CA3 and BLA brain areas revealed more evident co-labeling of α-subunits with Geph in non-AIS locations, suggesting the associations of α-subunits with Geph were stronger in non-AIS than AIS locations, **Figures 7A,C,E**. This visual impression was confirmed by both correlational and overlap analyses. Correlations between the signal intensities of α2-subunits and Geph were reliable in non-AIS locations for both the CA3 (*r* = 0.449 ± 0.040) and BLA (*r* = 0.490 ± 0.028). In the AIS, these correlations were weaker (CA3, *r* = 0.052 ± 0.046, BLA, *r* = 0.263 ± 0.033). Similarly, the correlations between α3-subunits and Geph in the BLA were reliable in non-AIS locations (*r* = 0.482 ± 0.034) but weaker on the AIS (*r* = 0.289 ± 0.053). These finding were in line with the quantification results from images presented in **Figures 7B,D,F**. The correlations coefficients from α-subunits and Geph aggregates were significantly larger in non-AIS locations in comparison to AIS locations (*p*s < 0.01, Bonferroni correction).

Because the size and density of Geph clusters can influence degree of co-localization with subunits (Sassoè-Pognetto et al., 2000; Sun et al., 2004), we compared these measures in the AIS and non-AIS locations. In sections co-labeled with α2-subunits, both the density and size of Geph clusters were comparable in the CA3 (Density: AIS = 22.3 ± 1.3, non-AIS = 24.7 ± 1.1; Size: AIS = 0.197 ± 0.025 μm^2^, non-AIS = 0.190 ± 0.022 μm^2^). Likewise, the density and size of Geph clusters in the BLA were similar (Density: AIS = 29.2 ± 1.9; non-AIS = 28.7 ± 1.6; Size: AIS = 0.212 ± 0.028 μm^2^, non-AIS = 0.211 ± 0.016 μm^2^). In BLA sections co-labeled with α3-subunits, analyses of images revealed no differences in Geph density (AIS = 30.8 ± 2.0; non-AIS = 27.5 ± 1.4); however, the size of AIS clusters were slightly larger than non-AIS clusters (AIS = 0.214 ± 0.026 μm^2^; non-AIS = 0.169 ± 0.008 μm^2^).

Mander’s M1 coefficients, denoting the fractions of α-subunit immunoreactivity overlapping with Geph immunoreactivity, were significantly smaller on the AIS locations when compared to non-AIS locations (*p*s < 0.01, Bonferroni-corrected comparisons). On the other hand, Mander’s M2 coefficients, denoting the fraction of Geph immunoreactivity overlapping with α-subunit immunoreactivity, were significantly larger on the AIS when compared to non-AIS locations (*p*s < 0.01, Bonferroni-corrected comparisons). The latter findings were likely due to the greater size and density of α-subunits aggregates on AIS versus non-AIS locations (Figures 3 and 4); the relatively large α-subunits aggregates were more likely to completely overlap of the smaller Geph clusters in AIS locations. Nonetheless, our results indicate that the association of α2- and α3-subunits with Geph was significantly weaker on AIS versus non-AIS locations.

## Discussion

Axonal initial segment is a crucial subcellular domain on neurons where action potential is initiated and plays a pivotal role in neuronal physiology (Stuart et al., 1997; Kole et al., 2008). Past studies have shown that GABA_A_Rs clusters are found on the AIS of principal neurons and that these receptors predominant receive axo-axonic synaptic input from GABAergic neurons that undoubtedly play an important role for controlling cell excitability and regulating action potential generation. While it is largely recognized that GABA_A_Rs containing α1- and α2-subunits form clusters on the AIS, α3-GABA_A_R subunits are also located on the AIS. At present, less is known about the AIS expression pattern of α3-subunits. To address this issue, we measured protein expression of α3-subunits, in addition to α1- and α2-subunits, in order to characterize the subunit composition of GABA_A_R clusters on the AIS among different brain areas.

### Variability in the Expression of AIS-Located α-Subunits

In general, the relative expression patterns and immunoreactive intensities of α-subunits in brain areas of this study correlated to that in rats and mice reported previously. In agreement with past studies, we observed moderate expression of all three subunits in the BLA and cortical areas, whereas relatively weak expression of α3-subunits was seen in hippocampal regions compared to α1- and α2-subunits. The co-labeling of AnkG with separate α-subunits generally support previous studies showing clusters of GABA_A_Rs containing the α2-subunits are preferentially targeted to the AIS compared to those containing the α1-subunit.

With the exception of the cortex, all other brain areas examined showed higher densities of α2-subunits in the AIS verses non-AIS locations. However, the CA1, CA3, and BLA also showed α2-clusters which were significantly larger in the AIS verses non-AIS locations. For these subunit, the combination of higher densities and larger clusters within AIS clusters were concomitant with a significant enrichment ratio. The combination of these factors also help to explain the significant enrichment of α3-subunits in the AIS of the BLA. The DG, PFC, and BLA showed higher AIS densities of α3-subunit in the AIS verses non-AIS locations. However, only the BLA contained α3-clusters which were significantly larger in the AIS together with a significantly higher enrichment ratio. Our analysis revealed that the densities of AIS-located α1-subunits were significantly greater in the CA1, CA3, and BLA, indicating that α1-subunits showed some degree of preferential targeting to the AIS in these brain regions. Comparisons of enrichment ratios across different brain areas revealed that the enrichment of α1-subunits in the CA3 was significantly greater than other brain regions. This result was likely due to the fact that the density of AIS-located α1-subunit was three times as great as non-AIS locations in the CA3, as opposed to 1.5–1.8 times in the CA1 and BLA.

Along with α-subunits, AIS clusters in the cortex and the hippocampus are known to contain γ2-subunits (Christie and De Blas, 2003; Wimmer et al., 2010; Muir and Kittler, 2014) and β2- and/or the β3-subunits (Nusser et al., 1995, 1996), suggesting that the GABA_A_Rs in the clusters we observed were pentamers made of α1/2/3, β2/3, and γ2 subunit assemblies. Previous studies have identified the co-localization of different α-subunit isoforms within AIS-located GABA_A_R clusters. Unknown is whether these clusters may be composed of GABA_A_Rs containing two different α-subunit isoforms or the same α-subunit isoform (Christie and De Blas, 2003). Because the subunit-specific antibodies used in this study were raised in the same species, it was technically difficult to compare their co-expression in the same sections by double-labeling. Also unclear is the degree to which AIS GABA_A_Rs participate in extrasynaptic (tonic) inhibition. In neuronal preparations, the manipulation of GABA concentrations believed to reflect ambient (tonic) levels *in vivo* do not activate AIS GABA_A_Rs (Rojas et al., 2011), suggesting AIS GABA_A_Rs mediate synaptic inhibition. Some studies have reported the existence of two distinct populations of pyramidal cells based on the presence of diffuse and/or discrete α2-clusters within the AIS (Brünig et al., 2002; Christie and De Blas, 2003). The presence of non-clustered diffuse labeling suggests that some AIS GABA_A_Rs in pyramidal cells may also mediate extrasynaptic inhibition. In the brain areas analyzed here, we observed well-defined α-subunit immunoreactivity which formed discrete puncta within AIS areas, thus appearing to be mainly synaptically localized. Discrete aggregates also appeared to locate on the perisomatic membranes, however, in the absence of a fluorescent membrane dye, these clusters were difficult to clearly ascertain and quantify.

### Enrichment of AIS-Located α2- or α3-Subunits in the BLA is Unaffected by the Genomic KO of α3- or α2-Subunits

Our quantitative results of AIS clusters in mutant mice lacking α2- or α3-GABA_A_Rs are consistent with past studies revealing that the associated receptor subunit does not accumulate to form aggregates (Studer et al., 2006; Winsky-Sommerer et al., 2008; Panzanelli et al., 2011). Previous observations in α3-KO mice indicates the loss of α3-subunits does not change the regional distribution of the α1-, α2-, or α5-subunits or result in the replacement by another α-subunits variant expressed in the same cell (Studer et al., 2006). Further, the distribution of α2-subunit is found to be unchanged in mice globally lacking the α5-subunit (Fritschy et al., 1998b). We also found the clusters of the α2-subunits showed characteristics comparable to WT C57BL/6J mice in the α3-KO mice. Together, these data indicate that the enrichment of AIS-located α2-subunits are unaffected by genomic KO of α3-subunits.

The effects of α2-KO on the distribution of other AIS α-subunits are more complex, because genetic deletion α2-subunits can cause compensatory alteration in other GABA_A_Rs subunits that might function to protect against α2-subunit loss. It is reported that α2-KO causes an increase in α3- and α4-subunits protein levels across the entire CNS, however, the replacement of α2-subunits by another subunit variant appears dependent on the regional and subcellular location. For example, Gabra2 deletion increases α5-subunit but not α3- or α4-subunit expression in the hippocampus (Panzanelli et al., 2011). While the consequences of α2-KO in the AIS are difficult to predict, our results clearly show α2-KO does not result in the loss of AIS-located α3-subunits in the BLA. Collectively, these findings suggest that the enrichment of α2- or α3-subunits in the AIS of BLA neurons is unaffected by the genomic KO of the α3- or α2-subunits, and is likely to be physiologically independent of one another.

### Both α2- and α3-GABA_A_Rs are Preferentially Targeted to the AIS in the BLA

From double-labeling experiments, we observed both α2- and α3-subunit clusters were preferentially enriched in the AIS of the BLA. We also found that α1-subunits were preferentially targeted to the AIS but the enrichment was not as great as α2- and α3-subunits. This unique pattern was not identified in other brain areas examined in this study. Past immunohistochemical studies have identified diffuse α2- and α3-subunit staining, presumed to depict the local aggregation of extrasynaptic receptors in the somata and dendrites (Fritschy and Mohler, 1995; Pirker et al., 2000; Marowsky et al., 2012). We found both diffuse and discrete staining patterns for as all subunits, suggesting receptors were located both synaptically and extrasynaptically. Our morphological analysis did not quantify diffuse staining, however, the α3-subunit expression appeared more dispersed in non-AIS locations, signifying that α3-subunits are preferentially located at extrasynaptic sites that mediate tonic inhibition (Marowsky et al., 2004, 2012)

Our result shows that α-subunits on the AIS locations receive heavy innervation of vGAT-positive terminals. As reported previously, there are at least two distinct population of parvalbumin-positive (PV+) cell types that target principal cells in the BLA (Bienvenu et al., 2012). PV+ basket cells mainly target somata and dendrites, whereas axo-axonic chandelier cells mostly make synaptic contacts on the AIS. Together, these results indicate that the innervation to the AIS-located α-subunits is provided by PV+ chandelier cells and is closely coupled with postsynaptic GABA_A_Rs.

### Is the Clustering of α2- and α3-Subunit on the AIS Independent of Geph?

To explore the association of Geph with GABA_A_R subunit aggregates, we quantified their overlap as well as their correlation within and between AIS and non-AIS locations of the BLA and CA3. We found the fraction of α-subunits aggregates overlapping with Geph were significantly larger in non-AIS compared to AIS locations, as were the correlations between the intensities of α-subunits and Geph aggregates. Differences in correlations are consistent with a number of studies reporting intensity of immunostaining for Geph is weaker in the AIS in comparison to the somata or dendrites despite similarities in the presence and intensity of various α-subunits aggregates (Brünig et al., 2002; Panzanelli et al., 2011; Fritschy et al., 2012). Together these findings imply that Geph might be playing a lesser role in the clustering and enrichment of α2- and α3-subunits on the AIS. Numerous studies have shown that various GABA_A_R subunit clusters co-localize with Geph at putative postsynaptic sites (Sun et al., 2004). Past studies have revealed postsynaptic α2- and α3-subunits clusters co-localize with Geph clusters in many brain regions (Sassoè-Pognetto et al., 2000). Similarly, In hippocampal dentate granule cells, Geph clusters more commonly co-localized with large α1- and γ2-subunit clusters but rarely with small ones (Sun et al., 2004).

While the density of GABA_A_Rs is a major a factor in determining synaptic strength (Chiou et al., 2011), the association of Geph with an α-subunit is mediated by numerous variables that control the synaptic accumulation of GABA_A_Rs, such as Geph phosphorylation (Bannai et al., 2009; Panzanelli et al., 2011; Flores et al., 2015) and palmitoylation (Dejanovic et al., 2014). Furthermore, there is considerable data showing that synaptic clustering of α-subunits are governed by different mechanisms that may or may not depend on Geph (Sassoè-Pognetto et al., 2011; Fritschy et al., 2012; Tretter et al., 2012). With respect to the α1-subunits, immunohistochemical analyses have shown that the dystrophin-glycoprotein protein complex contributes to stabilization of perisomatic postsynaptic α1-subunit clusters and neuroligins cell adhesion molecules which is apparently not dependent on Geph (Levi et al., 2004; Panzanelli et al., 2011). Evidence showing the absence of dystrophin-glycoprotein protein in the AIS (Panzanelli et al., 2011; Fritschy et al., 2012), suggests this absence may contribute to the overall weak AIS enrichment of α1-subunit clusters, as seen in the present study.

The key components and mechanisms involved in the postsynaptic clustering of α2- and α3-subunits remain less clear. In somatodendritic synapses, the adaptor protein collybistin is co-localized with a subset of Geph-positive synapses in many brain areas, including the hippocampus and BLA (e.g., Papadopoulos et al., 2007; Fritschy et al., 2012). Some evidence suggest that collybistin/Geph aggregates, along with and cell adhesion molecules, are associated with the maintenance of postsynaptic GABA_A_Rs with α2-subunits, but not those with α3-subunits (Papadopoulos et al., 2008; Poulopoulos et al., 2009; Saiepour et al., 2010). Other data suggest collybistin plays a more complex role in the recruitment Geph and α-subunit variants to postsynaptic sites that depend on unique subcellular factors (Fritschy et al., 2012; Tyagarajan and Fritschy, 2014). In addition, published data have revealed the cell adhesion molecule neurofascin is highly localized on the AIS of hippocampal neurons and may also play an important role in the stabilization of AIS-located GABA_A_Rs (Burkarth et al., 2007; Kriebel et al., 2011; Zonta et al., 2011). Future work will surely determine how these and other key scaffolding regulators influence the postsynaptic accumulation of α-subunit GABA_A_Rs variants in the AIS.

### Technical Limitations

As noted by others, differences among the intensity levels of antibodies cannot be taken as absolute differences in subunit abundance due to the unique properties of each antiserum and features of immunolabeling in distinct brain regions (Fritschy et al., 1998a). Nonetheless, we reasoned that the apparent uniform labeling of each antibody within a specific regions (same image) could be used to most accurately estimate the relative abundance and differential expression patterns among brain regions. As mention above, the “enrichment” of GABA_A_R subunits in the AIS was defined by comparing the density of antibody aggregation in the AIS to non-AIS ROIs that likely represent subunit aggregates on the dendrites segments of principle cells and inhibitory interneurons. The subfields and neuronal layers of the hippocampus and cortex are intrinsically different in cell type, dendritic phenotypes, and synaptic integration which contribute to the distinctive expression patterns of GABA_A_R subunits in different brain regions. Due to the unique subunit assemblies and subcellular expression patterns of GABA_A_Rs, it is likely that estimates of enrichment vary different neuronal layers and subfield domains.

## Conclusion

While it is largely recognized that GABA_A_Rs containing α1- and α2-subunits form clusters on the AIS, our results confirm that α3-subunits are also located on the AIS across many brain area. We also found the expression pattern of α1-, α2-, and α3-subunits in the AIS differed across different brain areas. The most prominent finding of our study is the predominant presence of α3-subunit on the AIS of BLA neurons. The detection of α3-subunits in the AIS of other brain areas also underscores the potential contribution of this subunit in axo-axonic synaptic input. As with perisomatic synapses, AIS-located synapses are thought to be important for synchronization of large populations of pyramidal neurons. Because the α-subunit composition determines activation and deactivation kinetics of GABA_A_Rs (Lavoie et al., 1997; Dixon et al., 2014) as well as the power and frequency of θ- and γ-oscillations (Bienvenu et al., 2012; Hines et al., 2013), differences of the composition of α-subunits on the AIS may contribute to variability in the synchronization of pyramidal neurons among brain regions. In addition, GABA_A_Rs containing α1-, α2-, α3-, or α5-subunits adjacent to the γ2-subunit mediate different effects of benzodiazepines (Sigel and Buhr, 1997; Marowsky et al., 2004; Rudolph and Knoflach, 2011), thus distinctive subunit assemblies on the AIS may contribute to the diverse effects of benzodiazepines in various brain regions. Further research designed to study the function and plasticity of axo-axonic synaptic input on the AIS will undoubtedly contribute to a better understanding of the mechanisms involved in regulating GABAergic transmission and the diversity of GABAergic inhibition mediated by different α-subunits in distinct brain regions and cell types.

## Author Contributions

Both YG and SH conducted immunohistochemistry experiments, statistical analyses, and wrote manuscript.

## Conflict of Interest Statement

The authors declare that the research was conducted in the absence of any commercial or financial relationships that could be construed as a potential conflict of interest.
